# Hepatocyte specific TIMP3 expression prevents diet dependent fatty liver disease and hepatocellular carcinoma

**DOI:** 10.1038/s41598-017-06439-x

**Published:** 2017-07-27

**Authors:** Viviana Casagrande, Alessandro Mauriello, Simone Bischetti, Maria Mavilio, Massimo Federici, Rossella Menghini

**Affiliations:** 1 Department of Systems Medicine, University of Rome Tor Vergata, 00133 Rome, Italy; 2 Department of Biomedicine and Prevention, University of Rome Tor Vergata, 00133 Rome, Italy

## Abstract

Non-alcoholic fatty liver disease (NAFLD) encompasses a broad spectrum of conditions, ranging from non-progressive bland steatosis to hepatocarcinoma. Tissue inhibitor of metalloproteinase 3 (Timp3) has a role in the pathogenesis of fatty liver disease associated with obesity and is silenced during metabolic disorders and liver cancer. We generated an hepatocyte-specific TIMP3 ‘gain-of-function’ mouse model under the control of the Albumin promoter (AlbT3) and investigated its effects during high-fat diet (HFD). After 16 weeks of HFD, TIMP3 overexpression significantly improved glucose metabolism, hepatic fatty acid oxidation and cholesterol homeostasis. In AlbT3 mice CYP7A1, MDR3 and MRP2 gene expressions were observed, consistent with higher bile acid synthesis and export. Next, to evaluate the role of A Disintegrin and Metalloproteinase 17 (ADAM17), a crucial target of TIMP3, in these processes, we created mice deficient in Adam17 specifically in hepatocyte (A17LKO) or in myeloid lineage (A17MKO), founding that only A17LKO showed improvement in liver steatosis induced by HFD. Moreover, both, AlbT3 and A17LKO significantly reduced diethylnitrosamine-initiated, HFD-promoted hepatic tumorigenesis assessed by tumor multiplicity and total tumor area. Taken together, these data indicate that hepatic TIMP3 can slow progression of NAFLD, and tumorigenesis, at least in part, through the regulation of ADAM17 activity.

## Introduction

Non-alcoholic fatty liver disease (NAFLD) represents a spectrum of liver damage ranging from simple steatosis to non-alcoholic steatohepatitis (NASH) and advanced fibrosis which can progress to cirrhosis with a high risk of liver failure and hepatocellular carcinoma^1^. NAFLD is mainly associated with obesity, diabetes, dyslipidemia, insulin resistance (IR) and oxidant stress, which are the main features of the metabolic syndrome^2^. The hallmark histological feature of NAFLD is the accumulation of fat in hepatocytes without signs of secondary hepatic fat accumulation. Hepatic lipid content is regulated by balancing hepatic uptake, synthesis, oxidation, and export^3^.

The consequent surplus of lipids in hepatocytes results in oxidant stress and lipotoxicity and promotes the activation of the inflammation via a variety of intra- and inter-cellular signaling mechanisms leading to fibrosis^4^.

Among all pro-inflammatory cytokines involved in the pathogenesis of steatohepatitis, Tumor Necrosis Factor-α (TNF-α) represents a predictor of NASH and has a role in development of every setting of NAFLD. TNF-α levels correlate with advanced stages and its enhanced expression has been demonstrated in patients with NAFLD/NASH^5^.

The primary enzyme that cleaves membrane-bound TNF-α is A Disintegrin and Metalloproteinase 17 (ADAM17), which belongs to the ADAM family. ADAM17 activity is post-translationally regulated by Tissue Inhibitor of Metalloproteinases 3 (TIMP3), its only known physiological inhibitor^6^. TIMP3 is a 24–27 kDa protein belonging to the family of TIMPs that binds to the extracellular matrix and participates in the modulation of inflammation, cellular migration, and proliferation. Previous studies reported that the TIMP3/ADAM17 pathway is involved in the control of glucose homeostasis and inflammation in both genetic and nutritional models of obesity in mice, as well as in patients with obesity-related type 2 diabetes (T2D)^7^. Decreased TIMP3 was implicated in inflammation and IR; hepatic steatosis and liver inflammation was observed in TIMP3 knockout animals fed a high-fat diet (HFD) whereas ADAM17 activity is significantly increased in the liver and white adipose tissue (WAT) of mice fed a HFD in association with the development of IR and hepatosteatosis^8^. The association of TIMP3 with liver steatosis is explained, but only in part by gut microbiome dysbiosis, suggesting that TIMP3/ADAM17 dyad exerts both broad and local effects to modulate immune and metabolic pathways^9^.

To understand whether liver specific effects of TIMP3/ADAM17 play a role in modulating steatosis onset independently from other broad systemic effects on immune system and other tissues, we have generated transgenic mice overexpressing TIMP3 specifically in the liver (AlbT3), as well as hepatocyte specific ADAM17 knockout mice (A17LKO) or myeloid lineage-specific ADAM17 knockout mice (A17MKO).

## Results

### Generation of AlbT3 transgenic mice

**Figure 1:**
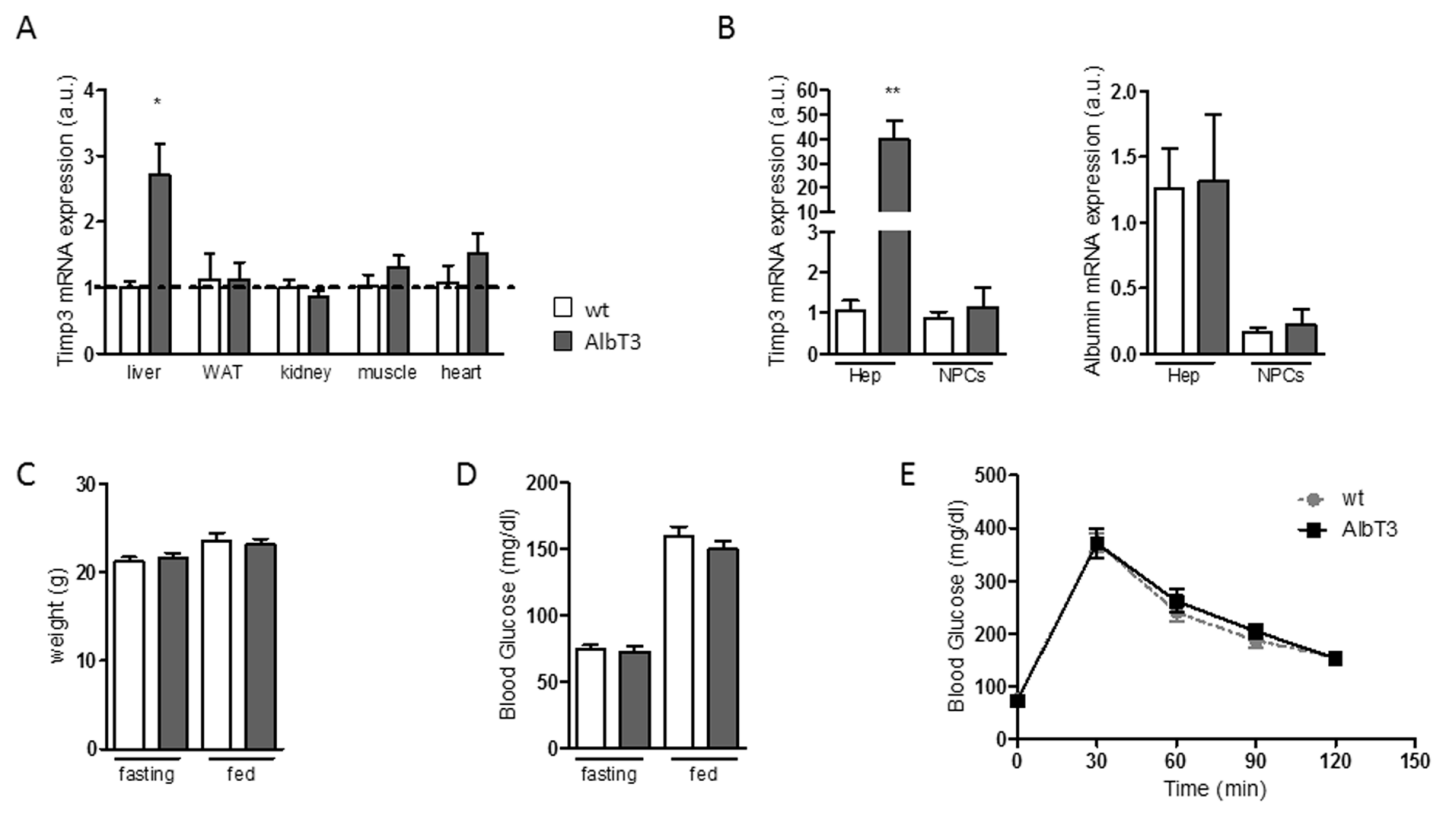
Generation and characterization of AlbT3 transgenic mice. (**A**) Relative amount of Timp3 mRNA expression in liver, WAT (with adipose tissue), kidney, muscle and heart of wild-type (wt) and AlbT3 mice. (**B**) TIMP3 and albumin (hepatocyte marker) mRNA expression in purified hepatocytes (Hep) and non-parenchymal cells (NPCs) from AlbT3 and wt mouse livers. Expression of mRNA was determined by qRT-PCR and values normalized to the endogenous control (β-actin). (n = 3 per group, *p < 0.03, **p < 0.01, Student’s t test, data are means ± SEM). (**C**) Weight and (**D**) blood glucose concentration. (**E**) Intraperitoneal glucose tolerance test (IPGTT). (n = 14 per group, data are means ± SEM). a.u.: arbitrary units.

To evaluate the effect of hepatic TIMP3 overexpression, we generated transgenic mice with targeted expression of Timp3 in hepatocytes (AlbT3) by using the well-established Albumin promoter-enhancer vector (Supplementary Fig. 1A,B)^10^. AlbT3 transgenic mice were viable, fertile and healthy and did not differ from wild-type (wt) mice in life span (data not shown). The tissue-specific overexpression of TIMP3 was confirmed by increased Timp3 mRNA level in AlbT3 liver homogenates but not in other metabolically active tissues such as adipose, kidney, skeletal muscle and heart compared with wt mice (Fig. 1A). In order to verify the cell-specific gene overexpression we analyzed Timp3 mRNA level in isolated hepatic tissue fraction, hepatocytes and non-parenchymal cells (NPCs), confirming that Timp3 mRNA was significantly overexpressed in AlbT3 hepatocytes (Fig. 1B). AlbT3 mice fed a chow diet do did not differ from wt littermates in terms of weight, fasting and fed glucose and glucose tolerance (Fig. 1C–E). Clinical chemical and hematological analyses did not reveal any abnormalities (data not shown).

### Metabolic effect of diet-induced obesity on AlbT3 mice

**Figure 2:**
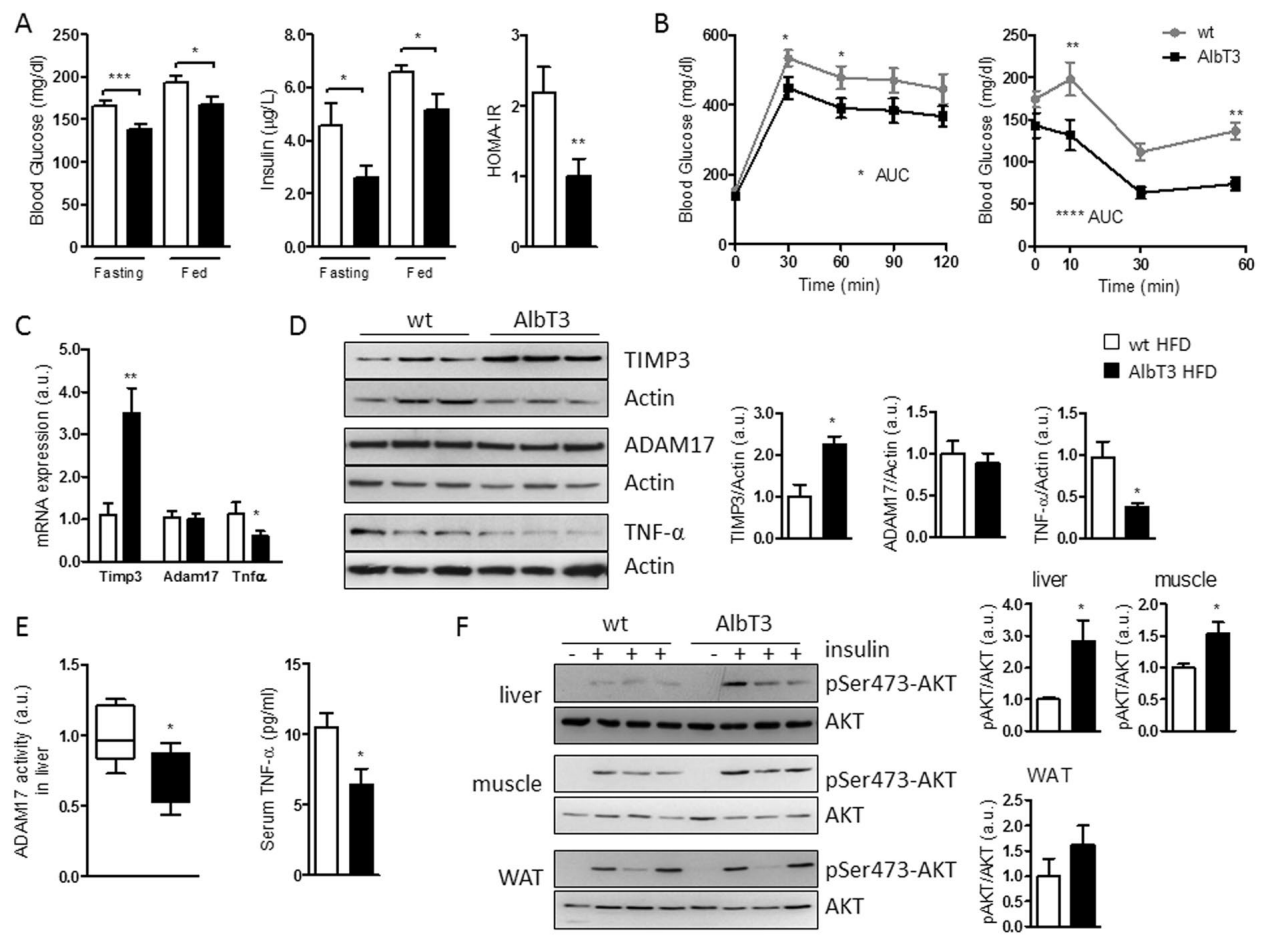
Metabolic effect in AlbT3 mice after 16 weeks of high-fat diet (HFD). (**A**) glucose (n = 20 per group); insulin levels and HOMA-IR (n = 8 per group). (**B**) IPGTT and intraperitoneal insulin tolerance test (IPITT) (n = 13 per group). (**C**) Timp3, Adam17 and Tnf-α mRNA expression (normalized to β-actin) and (**D**) TIMP3, ADAM17 and TNF-α protein levels in livers of AlbT3 and wt mice. (**E**) ADAM17 activity in livers and TNF-α levels in serum of wt and AlbT3 (n = 7 per group). (**F**) Akt phosphorylation (pSer473-Akt) in liver, skeletal muscle and WAT (n = 5 per group). A representative cropped image of 3 mice per group is shown. (*p < 0.05, **p ≤ 0.02, ***p ≤ 0.005, ****p < 0.002; Student’s t test, data are means ± SEM). AUC: area under the curve.

To investigate the effects of TIMP3 overexpression on hepatic homeostasis during obesity, we fed AlbT3 and wt mice a HFD for 16 weeks. At the end of the treatment blood glucose, insulin levels and HOMA-IR index (fasting blood glucose X fasting insulin/405) were significantly lower in AlbT3 compared to wt mice both, in fasting and fed conditions (Fig. 2A), although mice did not differ in weight (data not shown).

Similarly, AlbT3 mice showed improved glucose tolerance and insulin sensitivity compared with wt mice (Fig. 2B).

AlbT3 liver tissue showed significantly increased of TIMP3 and reduction of TNF-α expression, both at mRNA and protein levels, whereas mRNA and protein expression of ADAM17 is not affected (Fig. 2C,D). In addition, liver ADAM17 activity and serum soluble TNF-α (Fig. 2E) were significantly decreased in AlbT3 mice compared to wt, suggesting that, as expected, the liver overexpression of TIMP3 is able to regulate ADAM17 functions, rather than expression, during obesity.

Moreover, AlbT3 mice exhibited significantly improved phosphorylation of Ser473-Akt upon insulin stimulation in both liver and skeletal muscle tissues, whereas the WAT was unaffected (Fig. 2F).

**Figure 3:**
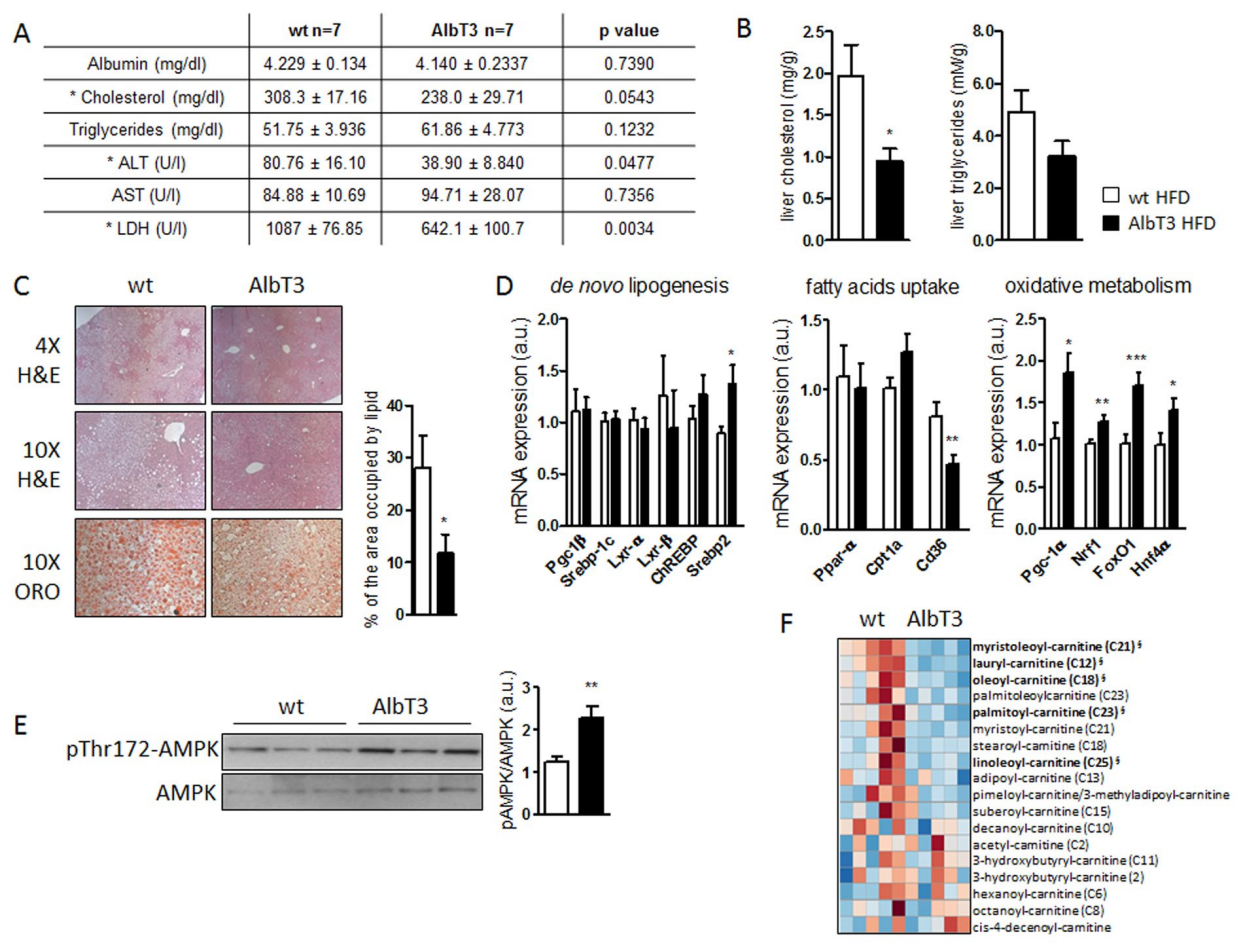
Reduced hepatic steatosis and cholesterol content in wt and AlbT3 mice after 16 weeks of HFD. (**A**) Serum biochemical analytes. ALT: alanine transaminase; AST: aspartate transaminase; LDH: lactate dehydrogenase (p values are shown). (**B**) Liver cholesterol and triglycerides content. (**C**) Representative pictures (×4 and ×10 magnification) of liver stained with hematoxylin/eosin (H&E) and Oil Red O (ORO). Quantification of the area occupied by lipid in ORO staining. (**D**) Liver mRNA expression of genes involved in *de novo* lipogenesis, fatty acids uptake and oxidative metabolism. (normalized to β-actin). (**E**) Representative cropped image of Western blots for AMPKα phosphorylation (pThr172-AMPKα) in liver extract. (n = 7 per group; *p < 0.05, **p ≤ 0.02, ***p ≤ 0.005; Student’s t test, data are means ± SEM). (**F**) Heatmap of acyl-carnitines in serum. Regions of red or blue indicate that the metabolite content is increased or decreased, respectively (n = 5; ^§^indicates significant difference with p ≤ 0.05 between the groups, metabolite ratio of <1.00; Welch’s Two-Sample t-Test).

Consistent with protection from liver damage, AlbT3 mice fed a HFD showed a significant decrease in the activities of functional enzymes, alanine transaminase (ALT), and lactate dehydrogenase (LDH) in serum, compared with wt mice (Fig. 3A). Additionally, TIMP3 overexpression in liver significantly decreased plasma and hepatic levels of total cholesterol, while no consistent effect on plasma and hepatic triglycerides was observed (Fig. 3A,B). Histological examination demonstrated a significant reduction of steatosis in the liver of AlbT3 HFD mice and Oil Red O (ORO) staining of lipids further confirmed a massive fat deposition in the livers of HFD fed wt mice compared to AlbT3 mice (Fig. 3C).

Genes related to lipid synthesis, transport and degradation in the liver were next analyzed. HFD has been known to activate a lipogenic response in liver tissue, however, no significant differences between wt and AlbT3 mice fed HFD, were found in the hepatic mRNA expression of enzymes involved in the *de novo* synthesis of fatty acids, including peroxisome proliferator activated receptor (PPAR)-γ coactivator (PGC)-1β, sterol regulatory element binding transcription factor (SREBP)-1c, liver X receptors (LXR)-α/β and carbohydrate responsive element binding protein (ChREBP). However, Timp3 overexpression leads to increased expression of Srebp2 (Fig. 3D).

Furthermore, in liver of AlbT3 mice were significantly suppressed mRNA levels of cluster of differentiation 36 (CD36), a transmembrane protein which plays a crucial role in fatty acids uptake (Fig. 3D), while the expression of enzymes involved in mitochondrial uptake of fatty acids, such as PPAR-α and carnitine palmitoyl-transferase (CPT)-1a, did not change. Moreover, an enhanced expression of PGC-1α, a master regulator of mitochondrial biogenesis and function, and its coactivator genes, nuclear respiratory factor 1 (NRF1), hepatocyte nuclear factor 4α (HNF4α), and forkhead box O1 (FoxO1) was observed in AlbT3 mice (Fig. 3D), suggesting an improvement in hepatic fatty acid oxidation.

We therefore also determined protein levels for phosphorylated AMP-activated protein kinase alpha (AMPKα) on threonine 172 (pThr172-AMPKα) in liver tissues. Protein pThr172-AMPKα determined by Western blot analysis increased significantly (Fig. 3E), whereas long-chain acyl-carnitine content, measured in sera of HFD mice through metabolomics approach, was reduced (Fig. 3F) in AlbT3 animals compared to controls.

### Effect of hepatic Timp3 on cholesterol export pathway

**Figure 4:**
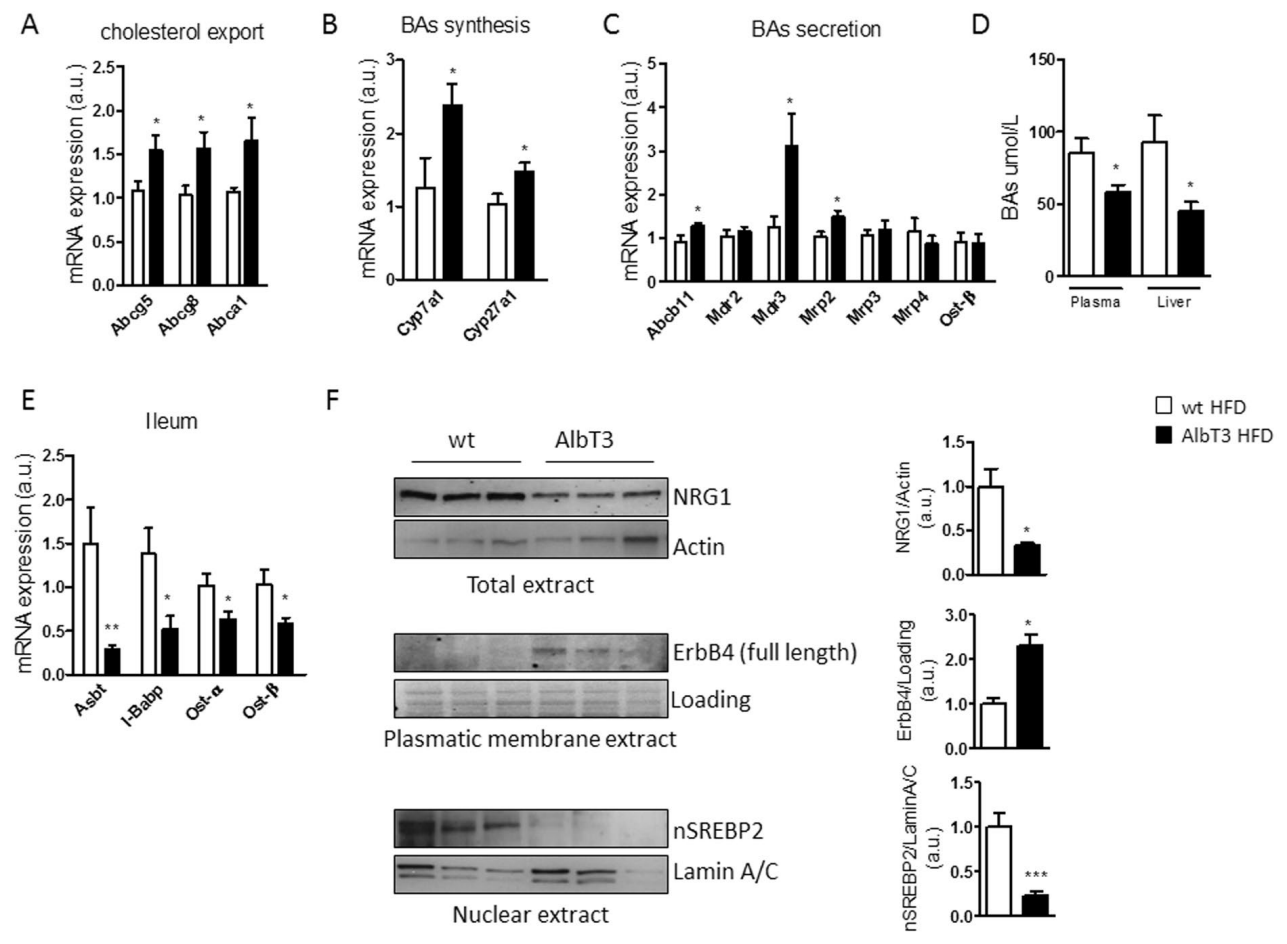
Bile acids (BAs) synthesis and transport regulation in wt and AlbT3 mice after 16 weeks of HFD. (**A,B,C**) Liver mRNA expression of genes involved in cholesterol export, BAs synthesis and secretion. (normalized to β-actin). (**D**) Plasmatic and hepatic total BAs levels. (**E**) mRNA expression of genes related to ileal BAs transporting, (normalized to β-actin). (**F**) Livers from wt and AlbT3 mice were analyzed for NRG1 in total extract (normalized to Actin), ErbB4 in plasmatic membrane extract (normalized to plasmatic membrane protein loading) and SREBP2 in nuclear extract (normalized to Lamin A/C) by Western blot; a representative cropped image of 3 mice per group is shown. (n = 7 per group; *p ≤ 0.05, **p ≤ 0.02, ***p < 0.01; Student’s t test, data are means ± SEM).

Enhanced cholesterol efflux from liver is a relevant mechanism to lower hepatic accumulation of lipids^11^. Cholesterol may be removed either as cholesterol itself or as bile acids (BAs)^12^. Genes related to cholesterol secretion, the membrane transporters ATP-binding cassette sub-family G member 5 and 8 (ABCG5, ABCG8), and ATP-binding cassette transporters A1 (ABCA1) resulted overexpressed in AlbT3 liver extract compared to wt (Fig. 4A), whereas ATP-binding cassette sub-family G member 1 (ABCG1) is reduced (Supplementary Fig. 2A). Next, we further investigated the BAs signaling in the regulation of cholesterol homeostasis in AlbT3 mice. AlbT3 mice showed increased mRNA expression of rate limiting enzymes in BAs synthesis, cholesterol 7α-hydroxylase (CYP7A1) and sterol 27α-hydroxylase (CYP27A1) compared to wt mice (Fig. 4B). Increased synthesis was accompanied, in AlbT3 mice, by a major amount of BAs secreted from hepatocytes into the canalicular lumen via the action of the bile salt efflux pump ATP-binding cassette B11 (ABCB11) (Fig. 4C).

Moreover, in AlbT3 mouse liver there was a significant increase in mRNA expression of multidrug resistance P-glycoprotein (MDR) 3, a special ABC-transporter necessary for the secretion of phospholipids into bile, and multidrug resistance protein (MRP) 2 which mediates the export of bilirubin conjugates. No differences in alternative basolateral BAs export mediated by MDR2, MRP3, MRP4 and organic solute transporters (OST) β were observed between the two groups (Fig. 4C). Overall these data suggest a preferential export of BAs secreted from hepatocytes into the canalicular lumen towards the gut.

Interestingly, AlbT3 mice still had about 2 fold lower plasma and hepatic total BAs levels than wt mice (Fig. 4D). Consistently with this result, genes related to BAs transporting inside ileal enterocytes such as the apical sodium-dependent bile salt transporter (ASBT), the intestinal BAs binding protein (I-BABP), OSTα and OSTβ were down-regulated in AlbT3 mice (Fig.4E).

Most of the free cellular cholesterol is located within the plasma membrane where levels are tightly regulated^13^. It has been shown that some epidermal growth factor receptors (EGFRs, also named ErbBs) could be part of a mechanism for sensing plasma membrane cholesterol concentration^14^. Recently it was proposed a model of ErbB4 activation on SREBP2/cholesterol signaling through ADAM17 and neuregulin-1 (NRG1) pathway. NRG1 activates ErbB4 and induced its proteolytic cleavage by ADAM17, resulting in an enhanced SREBP2 cleavage and nuclear activation^15^. In AlbT3 livers, compare to wt, we have found reduced NRG1 and increased uncleaved/full-length form of ErbB4 both at protein level (Fig. 4F). Consistently, in AlbT3 livers, despite Srebp2 mRNA was increased, and total protein level of SREBP2 did not change (Supplementary Fig. 2B), we have found a significantly reduction of nuclear protein content of SREBP2 (nSREBP2) (Fig. 4F) that may play a critical role in the activation of Cyp7a1 by impairing the ability of HNF4α to bind to its promoter^16^.

### Effect of hepatocyte and myeloid specific Adam17 deletion on glucose metabolism and liver steatosis

To evaluate whether the molecular mechanisms underlying the amelioration of hepatic metabolism by overexpression of TIMP3 in liver may depend, at least in part, on cell specific ADAM17 regulation, we generated both, hepatocyte-specific (A17LKO) and myeloid lineage-specific (A17MKO) ADAM17 knockout mice by crossing Adam^flox/flox^ ^17^ (Ct) mice with a Alb-Cre or LysM-Cre strains. Animals were born healthy and fertile, without any grossly apparent phenotypic differences.

Gene expression analysis revealed that, after 16 weeks of HFD, hepatic level of Adam17 mRNA was significantly lower in both models (Supplementary Fig. 3A). However, hepatic ADAM17 protein expression and activity decreased in A17LKO, but not in A17MKO mice compared with controls (Supplementary Fig. 3B,C). A17LKO showed a reduced ADAM17 activity in WAT (Supplementary Fig. 3C).

To confirm the specificity of the ADAM17 knockout in the A17LKO mice, hepatocytes and NPCs were isolated from the liver of HFD fed mice; quantitative real-time PCR (qRT-PCR) analysis showed a reduction of Adam17 mRNA expression in hepatocytes derived from A17LKO knockout compared to Ct mice (Supplementary Fig. 3D). For A17MKO mice characterization, we used NPCs cells subjected to a further separation with CD11b^+^ beads to enrich the monocyte compartment. NPCs-CD11b^+^ cells from A17MKO showed significantly lower ADAM17 expression compared to control mice (Supplementary Fig. 3E).

**Figure 5:**
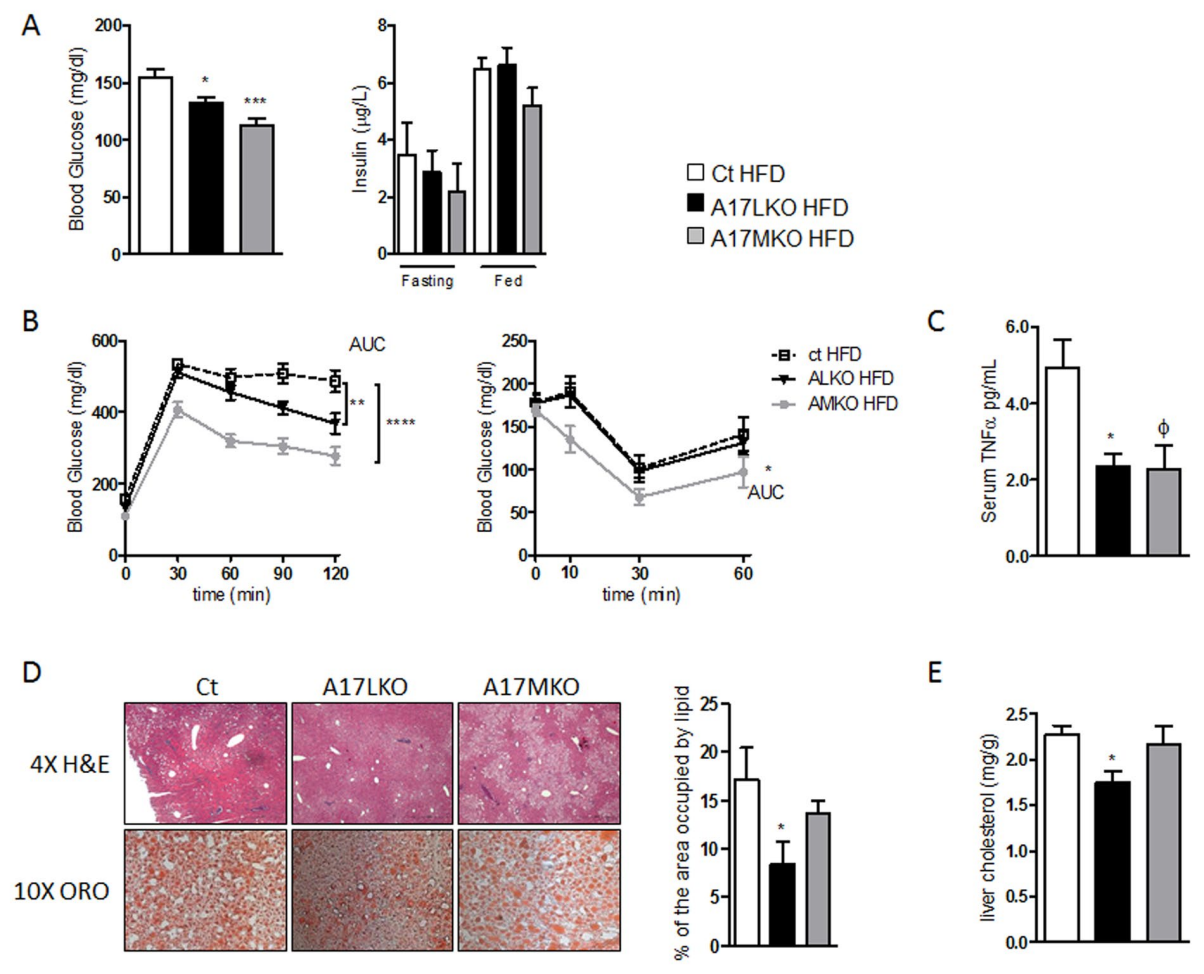
Effect of hepatocyte and myeloid specific Adam17 deletion on glucose metabolism and liver steatosis after 16 weeks of HFD. (**A**) Fasting blood glucose levels (n = 20 per group) and insulin levels (n = 6 per group). (**B**) IPGTT and ITT (n = 20 per group). (**C**) ELISA of serum TNF-α; (**D**) representative sections (×4 and ×10 magnification) of liver stained with H&E and ORO and quantification of the area occupied by lipid in ORO staining and (**E**) liver cholesterol content (n = 8 per group). (*p < 0.05, **p ≤ 0.02, ***p < 0.0005, ****p < 0.0001, φ = 0.0641; one-way ANOVA with Dunnett’s Multiple Comparison Test, data are means ± SEM).

Comparing the knockout mice with the controls, no differences were found in weight, cholesterol, triglycerides, ALT and LDH serum levels (data not shown) after 16 weeks of HFD. However, fasting blood glucose and glucose tolerance were significantly improved (Fig. 5A,B) in both specific knockout groups respect to control mice. A17MKO mice revealed also an improved insulin sensitivity compared with the other strains (Fig. 5B). Levels of TNF-α in serum were significantly reduced in A17LKO mice (Fig. 5C).

Hematoxylin/eosin and ORO staining revealed reduced steatosis only in A17LKO mice (Fig. 5D). This evidence was accompanied by a lower cholesterol level in liver extract (Fig. 5E), indicating that only hepatocyte specific deletion of ADAM17 has the protective effects on liver steatosis.

**Figure 6:**
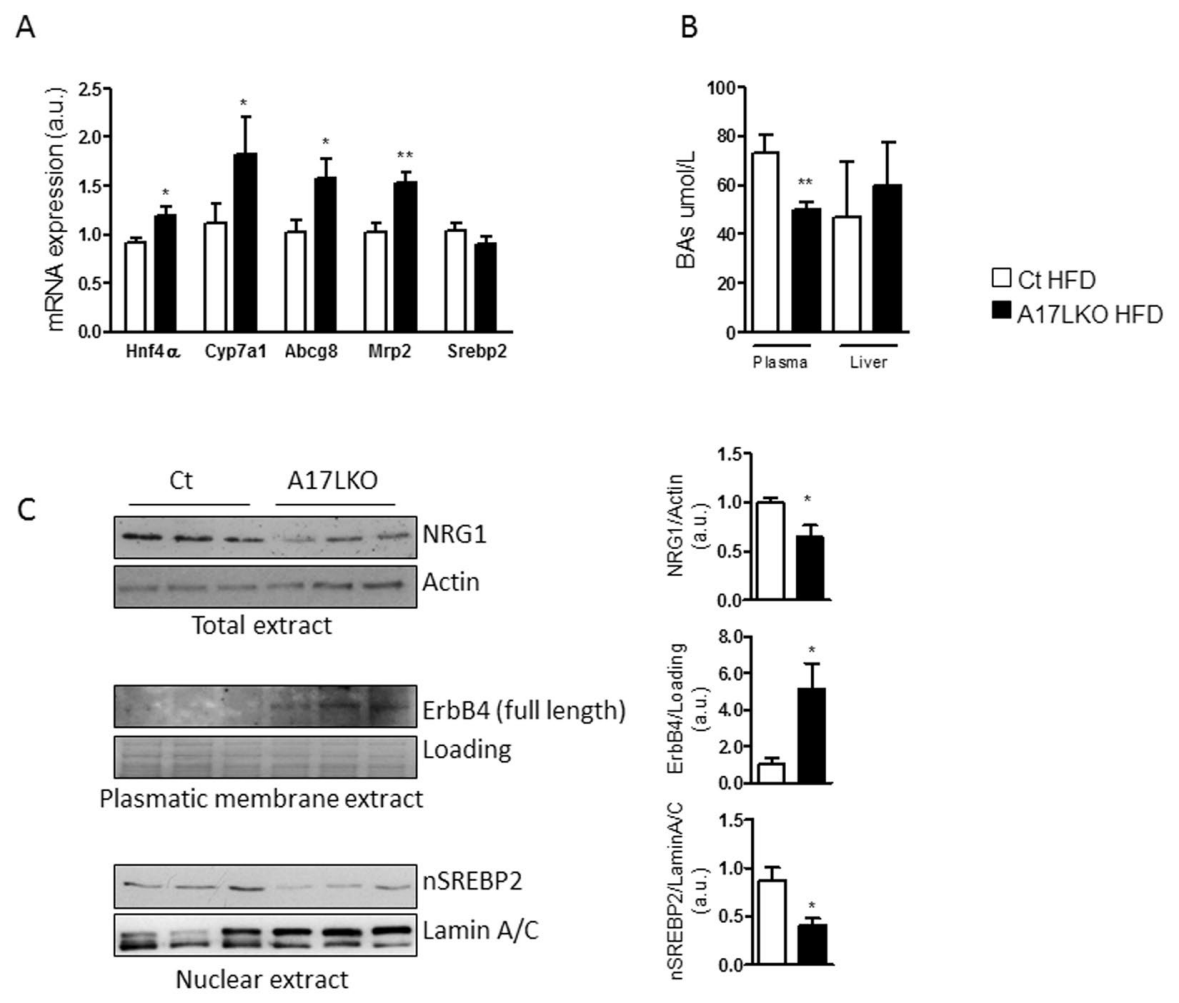
BAs regulation and ErbB4 signaling in A17LKO mice fed a HFD for 16 weeks. (**A**) Liver gene expression normalized to β-actin. (**B**) Plasma and liver content of total BAs. (**C**) Representative cropped image of Western blot for NRG1 in total extract (normalized to Actin), ErbB4 in plasmatic membrane extract (normalized to plasmatic membrane protein loading) and SREBP2 in nuclear extract (normalized to Lamin A/C) from livers of Ct and A17LKO mice. (n = 8 per group; *p < 0.05, **p < 0.02; Student’s t test, data are means ± SEM).

To recapitulate the major findings observed upon TIMP3 overexpression, we investigated the regulation of cholesterol export via BAs and ErbB4 signaling in A17LKO mice feeding a HFD. The main genes involved in the mechanism linking cholesterol/BAs/ErbB4 such as Hnf4α, Cyp7a1, Abcg8 and Mrp2, were increased in A17LKO similarly to AlbT3 mice (Fig. 6A). Moreover, A17LKO mice showed reduced plasmatic level of BAs compared with Ct (Fig. 6B) and Western blot analysis highlighted the significant reduction of NRG1 and nSREBP2 and the increased ErbB4 protein expression with the same trend as in AlbT3 mice (Fig. 6C).

### Effect of TIMP3/ADAM17 hepatocyte modulation on obesity related hepatocellular carcinoma

**Figure 7:**
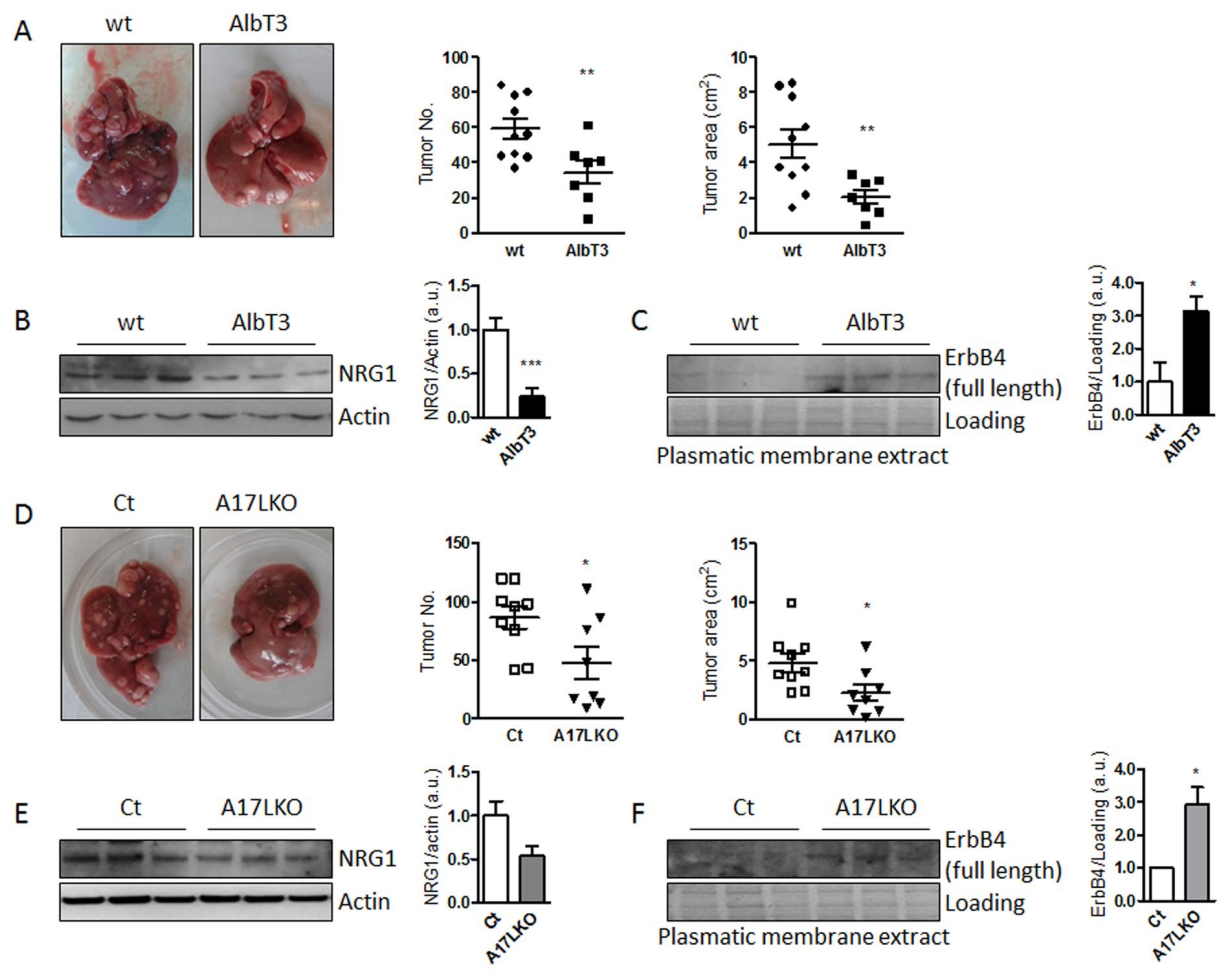
Timp3 overexpression or Adam17 selective deletion in hepatocyte reduced DEN-induced liver tumorigenesis during HFD. (**A**) Representative cropped images of wt and AlbT3 livers. The number and size of tumors in livers were counted. (**B**) NRG1 and Actin in total extract and (**C**) ErbB4 in plasmatic membrane extract analyzed by Western blot. (wt n = 10, AlbT3 n = 7). (**D**) Representative cropped images of Ct and A17LKO livers. The number and size of tumors in livers were counted. (**E**) NRG1 and Actin in total extract and (F) ErbB4 in plasmatic membrane extract analyzed by Western blot. (Ct n = 9, A17LKO n = 8). (*p < 0.05, **p < 0.02, ***p < 0.01; Student’s t test, data are means ± SEM).

Recent studies have identified TIMP3 as a tumor suppressor as its expression is silenced in malignant tumors including hepatocellular carcinoma (HCC)^18, 19^. To evaluate the role of hepatic Timp3 overexpression in HCC, we have used a model of diethylnitrosamine (DEN) administration combined with dietary induced obesity. After 7 months of HFD, Timp3 overexpression resulted in decreased HCC progression demonstrated by significantly reduced tumor number and size (Fig. 7A). Moreover, the NRG1 protein level was reduced and ErbB4 was increased in AlbT3 mice compared to wt (Fig. 7B,C). Since NRG1-ErbB4 pathway may activate intracellular signaling, leading to cell proliferation, survival, and differentiation^20^ we hypothesized that a protective role of TIMP3 in HCC may depend at least in part from the regulation of NRG1-ErbB4 signaling.

We performed the same protocol in A17LKO mice founding a significant protective effect on tumor number and size compared with controls (Fig. 7D) and a similar modulation of NRG1-ErbB4 signaling observed in AlbT3 (Fig. 7E,F).

## Discussion

TIMP3 affects extracellular matrix organization and participates to governing inflammatory cells trafficking in several tissues^7, 21^. Here, we describe that local hepatocyte specific overexpression of Timp3 impacts on glucose tolerance, insulin sensitivity and cholesterol metabolism and address potential mechanisms that explain these effects. Overexpression of Timp3 within the hepatic environment results in a promotion of lipid oxidative metabolism, supported by enhanced AMPK activation and reduction of acyl-carnitine levels. Accumulation of acyl-carnitines, byproducts of fat, glucose, and amino acid, is indicative of inefficient β-oxidation and mitochondrial dysfunction^22^ and it has been already known that amplified hepatic AMPK activation contributes to reduce hepatic lipid storage by increasing fatty acid oxidation with a major reduction in acyl-carnitine levels^23^. Another marked effect of hepatocyte Timp3 overexpression concerns cholesterol metabolism. In particular, AlbT3 mice manifest a trend favoring BAs export that is partially compensated by increased Srebp2 expression. The net results are decreased liver cholesterol content as well as liver and plasma BAs levels. Recently, it has been shown that subjects with T2D and obesity suffer from dysregulated lipids and BAs metabolism and that manipulation of the BAs pool can improve glycemic control in such patients^24^. Moreover, while in subjects with T2D several steps of cholesterol metabolism are altered^25^, in AlbT3 mice we found improvements within the pathway governing cholesterol utilization to synthetize BAs and their transport.

When challenged with overnutrition, AlbT3 mice phenocopies in part the Cyp7a1 transgenic (Cyp7a1-tg) mice^26, 27^. However, unlike Cyp7a1-tg, we did not found neither differences in weight between AlbT3 mice and wt under HFD nor on TGR5 levels in brown adipose tissue (data not shown) suggesting that the major metabolic effects in AlbT3 is not mediated by thermogenesis. Nevertheless, it should be noted that metabolic effect of Cyp7a1-tg was observed in female mice^26^ while we only studied male mice.

**Figure 8:**
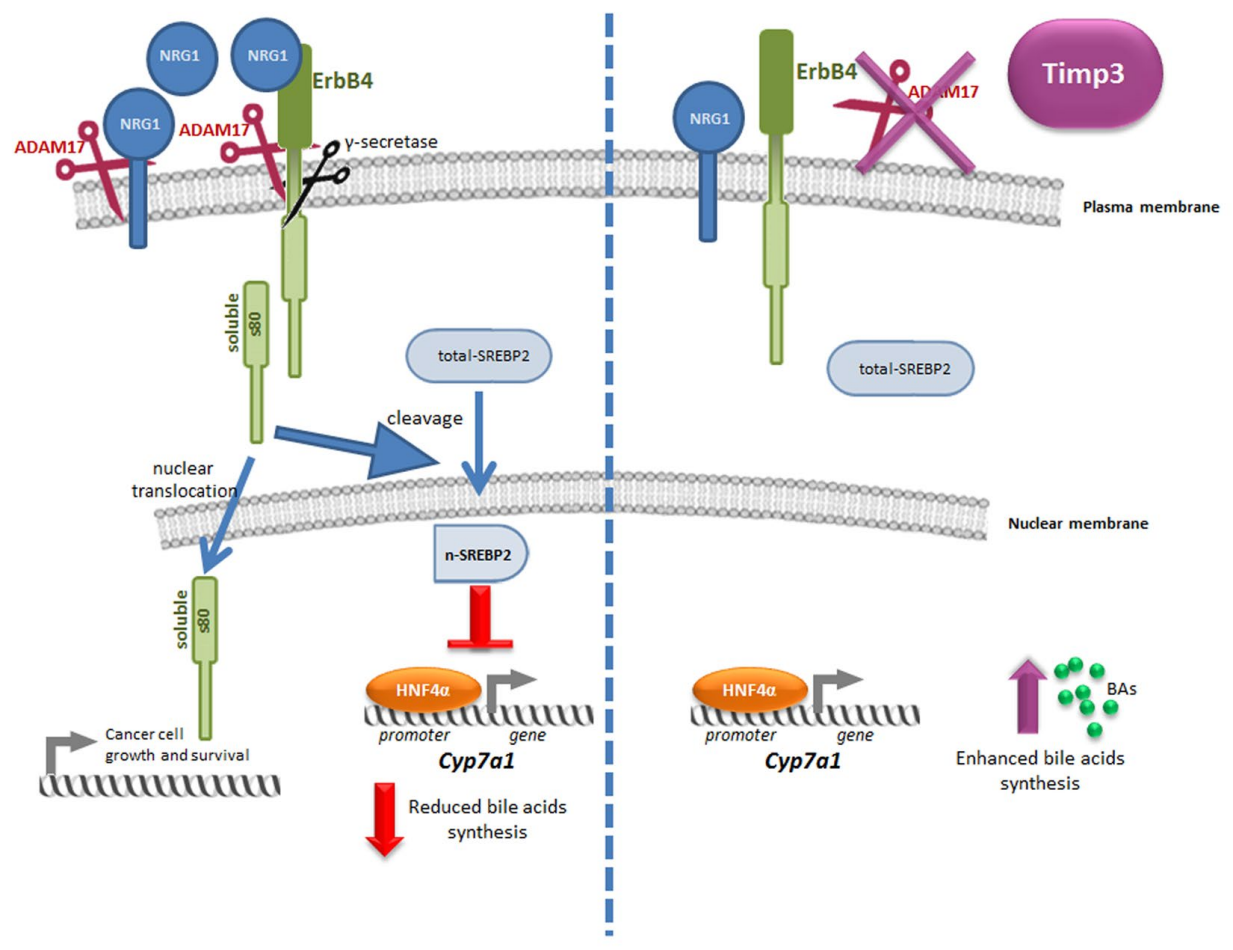
TIMP3 downregulates NRG1/ErbB4/SREBP2 signaling by the inhibition of ADAM17 activity, resulting in increased BA synthesis through enhancing Cyp7A1 expression.

Modulation of ADAM17 in hepatocyte and myeloid compartments in part recapitulates the effect of Timp3. This is explained by effects of TIMP3 on multiple proteases and receptors, suggesting that its metabolic effects are only in part due to ADAM17 inhibition and posing TIMP3 reconstitution at a more advanced stage as an approach against metabolic diseases compared to ADAM17 inhibition. One aspect that will deserve future investigation relates to the vascular bed as mediator of TIMP3 metabolic effects independently from ADAM17. Among factors that may underlie the mechanistic effects of TIMP3 we observed the modulation of NRG1 and its receptor ErbB4. ErbB4 belongs to the EGFRs family and is regulated by ADAM17^28^. In response to NRG1 binding to ErbB4, ADAM17 cleaves ErbB4 extracellular domain from the full-length protein, leaving the membrane-anchored m80 form. ErbB4 m80 may therefore undergo intramembrane cleavage by γ-secretases to release the soluble s80 form comprising the intracellular domain^20, 29^. s80 is able to bind transcriptional co-regulators and transcription factors, including Srebp2, enhancing nuclear translocation^15^. Given that AlbT3 mice showed reduced NRG1 expression and nuclear accumulation of SREBP2 it is intriguing to hypothesize that Timp3 acts on the NRG1/ErbB4/SREBP2 pathway to reduce cholesterol accumulation inside cells. In fact, during HFD conditions TIMP3 overexpressed in hepatocytes inhibits ADAM17 functions limiting NRG1 and ErbB4 activation and resulting in reduced nuclear amount of Srebp2 protein. The nuclear form of SREBP2 contributes to impair the ability of HNF4 orphan nuclear receptor to activate the Cyp7A1 transcription^16^. The interference of TIMP3 overexpression on this signaling pathway may lead to an increase of bile acid synthesis and cholesterol efflux explaining in part our observations (Fig. 8). Furthermore, it has been proposed that even TNF-α, that we found reduced in serum and liver tissue of AlbT3 and A17LKO models might contribute to the decline of Cyp7a1 transcription, mimicking the effects of BAs, via HNF4α regulation^30^. Consistently, we found increased expression of HNF4α targets such as phosphoenolpyruvate carboxykinase 1 (Pck1), microsomal triglyceride transfer protein (Mttp) e Lipin1^31, 32^ in both AlbT3 and A17LKO models (Supplementary Fig. 4). In this context, we can hypothesize that TIMP3 downregulates the fraction of nSREBP2 at a threshold that allows Srebp2 transcription but prevents its blocking effect on HNF4α transcription factor and, consequently, on Cyp7a1, contributing to maintain the homeostasis of cholesterol and BAs, crucial regulators of lipid, glucose and energy metabolism.

Because NASH is a risk factor for HCC, a type of cancer increased in obesity^33^ we tested whether the protective effects of hepatocyte specific Timp3 overexpression and at a less extent of ADAM17 deletion will result in a slower tumor progression using a previously validated model^34^.

Interestingly, different cancer tissues and cell lines seem to predominantly express cleavable ErbB4 isoforms^35^. Both AlbT3 and A17LKO mice showed in the tumor tissue increased expression of the unclipped ErbB4 isoforms and decreased level of NRG1 suggesting that the downregulated signals mediated by this are associated to decreased tumor progression and areas.

Our results are partially at odds with recently reported anti-tumoral effects of Timp3 knockout on HCC^36^. However, it is impossible to exclude that fetal effects of Timp3 loss may counteract DEN action independently by its effects in adult life, especially because DEN promotes tumors in a very early post-natal life^37^. Furthermore, the effects of TIMP3 on cancer progression appeared to be context dependent in several studies^38, 39^. However, in HCC studies TIMP3 expression was generally reported as decreased and among genes involved in a favorable survival in experimental models^19, 40, 41^. Consistently, it has been reported that ADAM17 activity is increased in HCC^42^.

In conclusion, we present evidence that TIMP3 functions as a protective metabolic sensor in the liver under nutritional induced obesity, preventing hepatic lipid accumulation and hepatocarcinogenesis. Hepatocyte specific overexpression of TIMP3 decreases plasma and liver cholesterol, enhances FFA oxidation and improves BAs export. In particular, we found that TIMP3 may exert hepato-protective actions via AMPK activation and NRG1/ADAM17/ErbB4 pathway, two signals involved on the modulation of Srebp2 nuclear accumulation. Therefore, strategies to increase TIMP3 activity specifically in hepatocytes may be explored as a valuable therapeutic approach to attenuate NAFLD development and liver tumor progression.

## Methods

### Animal models

Transgenic mice with targeted expression of Timp3 in the liver were developed by using Albumin promoter-enhancer driven vector^34^. To construct the Albumin promoter-Timp3 transgene, a 650 bp murine Timp3 cDNA with polyadenylation site (200 bp) was inserted in a vector containing Albumin promoter/enhancer and amplified in Escherichia coli strain DH5-α. The construct was linearized with endonuclease enzymes (SstII) and was micro-injected into mouse zygotes using standard methods. Offspring derived from the injections were genotyped by PCR analysis on DNA isolated from tail biopsies performed with primers that amplified a 850 bp fragment of the transgenic construct. The AlbT3 transgenic mice used in this study were derived from transgenic line that was backcrossed to C57Bl/6 wt mice for five consecutive generations.

Homozygous Adamflox/flox^17^ (referred to as Ct) were mated with B6.Cg-Tg(Alb-cre)21Mgn/J (referred to as Alb-Cre) or with B6.129P2-Lyz2tm1(cre)Ifo/J (referred to as LysM-Cre) to generate specific ADAM17-deficient mice (A17LKO and A17MKO respectively). In all experiments with Adamflox/flox/Cre mice, Ct mice served as controls. B6.Cg-Tg(Alb-cre)21Mgn/J and B6.129P2-Lyz2tm1(cre)Ifo/J mice were purchased from The Jackson Laboratory (Bar Harbor, ME). C57Bl6 mice were purchased from Charles River Laboratories (Wilmington, MA).

Mice were fed ad libitum with standard laboratory chow (10% calories from fat; Mucedola s.r.l., Settimo Milanese, Italy) or HFD (60% of calories from fat; Research Diets, New Brunswick, NJ) for 16 weeks after weaning and had free access to water. For AlbT3 characterization were used 8 weeks old male mice feeding a standard diet.

The handling of mice and experimental procedures were conducted in accordance with experimental animal guidelines. Male C57Bl6, AlbT3, Ct, A17LKO and A17MKO mice were maintained in our animal facility (12 h light/dark cycle; 22 °C ± 1 °C, 50% ± 5% humidity). At the end of each experiment, mice were sacrificed by CO2 asphyxiation. Animal studies were approved by the University of Tor Vergata Animal Care and Use Committee.

### Metabolic tests

Metabolic testing procedures for glucose homeostasis (blood glucose measurement, glucose tolerance test, insulin tolerance test, insulin signaling studies) have been described previously^34^.

Hormones and cytokine levels in serum were measured using commercial kits: insulin (Mercodia, Uppsala, Sweden), TNF-α (R&D Systems, Minneapolis, MN).

For determination of clinical chemistry parameters, blood samples from overnight-fasted mice were collected in Serum Separator Tube microtainers (Becton, Dickinson and Company, Franklin Lakes, NJ) and centrifuged at 13000 rpm for 7 min to separate the serum. Serum albumin, cholesterol, triglycerides, alanine transaminase (ALT) and lactate dehydrogenase (LDH) were measured using the automatic analyzer Keylab (BPC BioSed s.r.l., Rome, Italy).

Cholesterol and triglycerides extracted from liver tissues were analyzed using the Total cholesterol Assay Kit and Triglyceride Quantification Kit (Abcam, Cambridge, UK) according to the manufacturer’s instruction.

Total bile acids in serum and liver were determined with a bile acid assay kit (Crystal Chem, Downers Grove, Il) in overnight-fasted mice. Serum samples were used directly. Livers were homogenized at room temperature in 1 ml of 75% ethanol and incubated 2 h at 50 °C in glass tubes, then centrifuged at 6000 g for 10 minutes at 4 °C. Total bile acids were analyzed in supernatant fraction as described by manufacturer’s instruction.

### Metabolomics

For metabolomics analysis of acyl-carnitines, the serum was collected in fasting state after 16 weeks of HFD. Metabolomics assay was performed in service at Metabolon Laboratory as described in Supplemental Materials.

### Isolation of cells from liver

Primary hepatocytes and NPCs were isolated from livers as previously described^7^. Further separation was performed in A17MKO and Ct mice non-parenchymal fraction. A single cell suspension of non-parenchymal fraction was obtained passing cells through 30 µm nylon mesh and centrifuged at 300 g for 10 minutes. The pellet was resuspended in a degassed buffer (PBS pH7.2, 0,5% BSA, 2 mM EDTA) and subsequently CD11b MicroBeads (Miltenyi Biotec, Bergisch Gladbach, Germany) were added as indicated by the manufacturer. Selection of CD11b^+^ and CD11b^−^ was performed using large cell separation columns (Miltenyi Biotec).

### Gene expression analysis

Total RNA was isolated from tissue homogenates (liver, WAT, kidney, skeletal muscle, heart, ileum) and hepatocytes and NPCs from livers; qRT-PCR was performed as described in Supplemental Materials.

### Tissue lysis and Western blots

Western blots were performed on total tissue homogenates and nuclear extracts prepared as previously described^34^.

For preparation of membrane protein fraction, livers were minced into small pieces and homogenized in cold buffer (250 mM sucrose, 1 mM EDTA, 10 mM Tris HCl buffer, pH7.2 plus protease inhibitors). Nuclei and cell debris were removed by centrifugation at 500 g for 10 min at 4 °C, supernatants were subjected to high-speed centrifugation at 100000 g, 4 °C, for 1 h. Pellets were washed with homogenization cold buffer and recentrifuged at 100000 g, 4 °C, for 1 h. The pellet contains membrane fraction.

The lysates were subjected to SDS-PAGE and immunoblot analysis. The following antibodies were used: TIMP3, TNFα, ADAM17, SREBP2 (Abcam), anti–phospho-Ser473 Akt, total Akt, anti–phospho-Thr172 AMPK, total AMPK, ErbB4 (Cell Signaling Technology, Danvers, MA), NRG1 (Genetex, Irvine, CA), Lamin A/C, Actin (Santa Cruz Biotechnologies, Dallas, TX).

### ADAM17 activity

ADAM17 activity was determined as previously described^8^.

### Histology of liver sections

Histology was performed as previously described^34^. Briefly, the livers were fixed in 4% paraformaldehyde and embedded in paraffin, 10 μm–thick sections were cut and stained with H&E for routine morphology.

Oil Red O Staining was performed in OCT embedded tissue. Liver tissues were embedded in cryostat embedding medium and frozen. Frozen tissues were cut into 10-μm-thick cryosections and stained with Oil Red O.

The area occupied by lipid was measured using computer-assisted image analysis software ImageJ.

### Hepatocarcinoma induction

25 mg/kg DEN was injected intraperitoneally into 14 days old male mice according to an established protocol^37^. After 4 weeks, mice were fed HFD until sacrifice at 36 weeks of age. Tumors in each liver lobe were counted and measured.

### Statistical analysis

Results of the experimental studies are expressed as means ± standard error of the mean (SEM). Statistical analyses were performed using GraphPad Prism (v.5.02). Groups were compared using a two-tailed unpaired Student’s t test, Welch’s t-Test or one-way ANOVA with Dunnett’s Multiple Comparison Test as indicated. Values of p < 0.05 were considered to be statistically significant.

## Electronic supplementary material

Supplemental Information
