# Supplementary material for: Hepatocyte specific TIMP3 expression prevents diet dependent fatty liver disease and hepatocellular carcinoma
Source: Sci Rep. 2017 Jul 27;7:6747. doi: 10.1038/s41598-017-06439-x (PMC5532242; doi:10.1038/s41598-017-06439-x)
Supplement: Supplementary file 1 — Supplemental Information [file 41598_2017_6439_MOESM1_ESM.pdf]

Hepatocyte specific TIMP3 expression prevents diet dependent fatty liver disease and hepatocellular carcinoma

Viviana Casagrande[1], Alessandro Mauriello[2], Simone Bischetti[2], Maria Mavilio[1], Massimo Federici[1], Rossella Menghini[1]

[1]Department of Systems Medicine, University of Rome TorVergata, 00133 Rome, Italy

[2]Department of Biomedicine and Prevention, University of Rome TorVergata, 00173 Rome, Italy

Contents:

1. Supplementary methods
2. Supplementary Figure Legends
3. Supplementary Figures
4. Supplementary Full length Blots and Gels

1. Supplementary Methods

Gene expression analysis: Total RNA was prepared from total tissue homogenates and cells using TRIzol reagent (Life technologies, NY) according to the manufacturer's protocol. A total of 2 mg total RNA was reverse transcribed into cDNA using the High Capacity cDNA Archive kit (Applied Biosystems, Foster City, CA). Quantitative real-time polymerase chain reactions (qRT-PCR) were performed on individual samples using an ABI PRISM 7700 System and TaqMan reagents (Applied Biosystems). Each reaction was performed in triplicate using standard reaction conditions: 1 cycle at 50°C for 2 min, 1 cycle at 95°C for 10 min, and 40 cycles each at 95°C for 15 sec and 60°C for 1 min. Calculations were performed via a comparative cycle threshold method.

Metabolomic analysis: Metabolomic analysis Sample Accessioning: each sample received was accessioned into the Metabolon LIMS system and was assigned by the LIMS a unique identifier that was associated with the original source identifier only. This identifier was used to track all sample handling, tasks, results etc. The samples (and all derived aliquots) were tracked by the LIMS system. All portions of any sample were automatically assigned their own unique identifiers by the LIMS when a new task is created; the relationship of these samples is also tracked. All samples were maintained at -80°C until processed.

Sample Preparation: samples were prepared using the automated MicroLab STAR® system from Hamilton Company. A recovery standard was added prior to the first step in the extraction process for QC purposes. Sample preparation was conducted using aqueous methanol extraction process to remove the protein fraction while allowing maximum recovery of small molecules. The resulting extract was divided into four fractions: one for analysis by UPLC/MS/MS (positive mode), one for UPLC/MS/MS (negative mode), one for GC/MS, and one for backup. Samples were placed briefly on a TurboVap® (Zymark) to remove the organic solvent. Each sample was then frozen and dried under vacuum. Samples were then prepared for the appropriate instrument, either UPLC/MS/MS or GC/MS.

Ultrahigh performance liquid chromatography/Mass Spectroscopy (UPLC/MS/MS): the LC/MS portion of the platform was based on a Waters ACQUITY ultra-performance liquid chromatography (UPLC) and a

Thermo-Finnigan linear trap quadrupole (LTQ) mass spectrometer, which consisted of an electrospray ionization (ESI) source and linear ion -trap (LIT) mass analyzer. The sample extract was dried then reconstituted in acidic or basic LC-compatible solvents, each of which contained 8 or more injection standards at fixed concentrations to ensure injection and chromatographic consistency. One aliquot was analyzed using acidic positive ion optimized conditions and the other using basic negative ion optimized conditions in two independent injections using separate dedicated columns. Extracts reconstituted in acidic conditions were gradient eluted using water and methanol containing 0.1% formic acid, while the basic extracts, which also used water/methanol, contained 6.5mM Ammonium Bicarbonate. The MS analysis alternated between MS and data-dependent MS2 scans using dynamic exclusion. Raw data files are archived and extracted as described below.

Gas chromatography/Mass Spectroscopy (GC/MS): the samples destined for GC/MS analysis were re-dried under vacuum desiccation for a minimum of 24 hours prior to being derivatized under dried nitrogen using bistrimethyl-silyl-triflouroacetamide (BSTFA). The GC column was 5% phenyl and the temperature ramp was from 40°C to 300°C in a 16 minute period. Samples were analyzed on a Thermo-Finnigan Trace DSQ fast-scanning single-quadrupole mass spectrometer using electron impact ionization. The instrument was tuned and calibrated for mass resolution and mass accuracy on a daily basis. The information output from the raw data files was automatically extracted as discussed below.

Quality assurance/quality control: for QA/QC purposes, additional samples were included with each day's analysis. These samples included extracts of a pool of well-characterized human plasma, extracts of a pool created from a small aliquot of the experimental samples, and process blanks. QC samples were spaced evenly among the injections and all experimental samples were randomly distributed throughout the run. A selection of QC compounds was added to every sample for chromatographic alignment, including those under test. These compounds were carefully chosen so as not to interfere with the measurement of the endogenous compounds. Data extraction and compound identification: raw data was extracted, peak-identified and QC processed using Metabolon's hardware and software. These systems are built on a web-service platform utilizing Microsoft's .NET technologies, which run on high-performance application servers and fiber-channel storage arrays in clusters to provide active failover and load-balancing (Dehaven et al.,

2010; Evans et al., 2009; Reitman et al., 2011). Compounds were identified by comparison to library entries of purified standards or recurrent unknown entities. Metabolon maintains a library based on authenticated standards that contains the retention time/index (RI), mass to charge ratio (m/z), and chromatographic data (including MS/MS spectral data) on all molecules present in the library. Furthermore, biochemical identifications are based on three criteria: retention index within a narrow RI window of the proposed identification, nominal mass match to the library +/- 0.2 amu, and the MS/MS forward and reverse scores between the experimental data and authentic standards. The MS/MS scores are based on a 2 comparison of the ions present in the experimental spectrum to the ions present in the library spectrum. While there may be similarities between these molecules based on one of these factors, the use of all three data points can be utilized to distinguish and differentiate biochemicals. More than 3500 commercially available purified standard compounds have been acquired and registered into LIMS for distribution to both the LC and GC platforms for determination of their analytical characteristics.

2. Supplementary Figure Legends

Supplementary Figure 1. (A) Integration of the transgenic construct into the mouse genome confirmed by polymerase chain reaction (PCR) analysis. (B) The number of transgene copies integrated in genome estimated by PCR. Ctrl: control. F1, F2: founder mice of AlbT3 colony. pAlb-T3-PolyA: construct containing Albumin promoter-Timp3 transgene-Polyadenylation site.

Supplementary Figure 2: (A) Abcg1 mRNA expression and (B) SREBP2 protein level in whole cell protein extracts (a representative cropped image of 3 mice per group is shown), determined in liver of AlbT3 and wt mice after 16 weeks of a HFD. mRNA and protein expression was normalized to β-actin (n=7 per group; **p<0.01, Students 't test, data are means ± SEM).

Supplementary Figure 3: Characterization of A17LKO and A17MKO mice after 16 weeks of a HFD. (A) Adam17 mRNA expression determined in liver. (B) ADAM17 protein level in liver and (C) ADAM17 activity in liver and in WAT. A representative cropped image of 4 mice per group is shown. (n=12 per group; *p<0.04, **p<0.01, ***p<0.0003, one-way ANOVA with Dunnett's Multiple Comparison Test, data are means ± SEM). (D) Adam17 mRNA expression in purified hepatocytes and NPCs from Ct and A17LKO mouse livers. Albumin mRNA expression was used as hepatocyte marker. (E) Adam17 mRNA expression in isolated $CD11b^+$ and $CD11b^-$ cells from non-parenchymal fraction from Ct and A17MKO mouse livers. mRNA expression was normalized to β-actin (n=4 per group; **p≤0.01, ***p<0.0003 Students 't test, data are means ± SEM).

Supplementary Figure 4. Liver expression of HNF4α targets in (A) wt and AlbT3 (n=7 per group), and (B) Ct and A17LKO (n=8 per group) mice on HFD. Expression of mRNA was determined by qRT-PCR and normalized to β-actin. (*p<0.05, **p≤0.02; Student's t test, data are means ± SEM).

3. Supplementary Figures

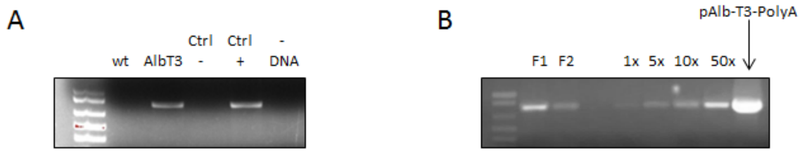

Suppl. 1

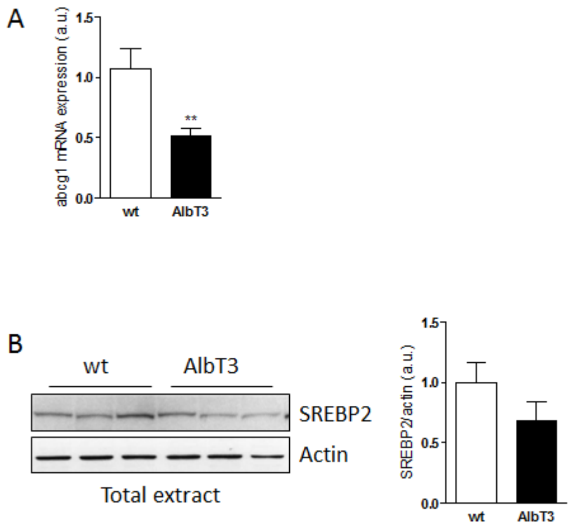
A
abcg1 mRNA expression (a.u.)
1.5
1.0
0.5
0.0
**
wt
AlbT3
B
wt
AlbT3
SREBP2
Actin
Total extract
SREBP2/actin (a.u.)
1.5
1.0
0.5
0.0
wt
AlbT3

Suppl. 2

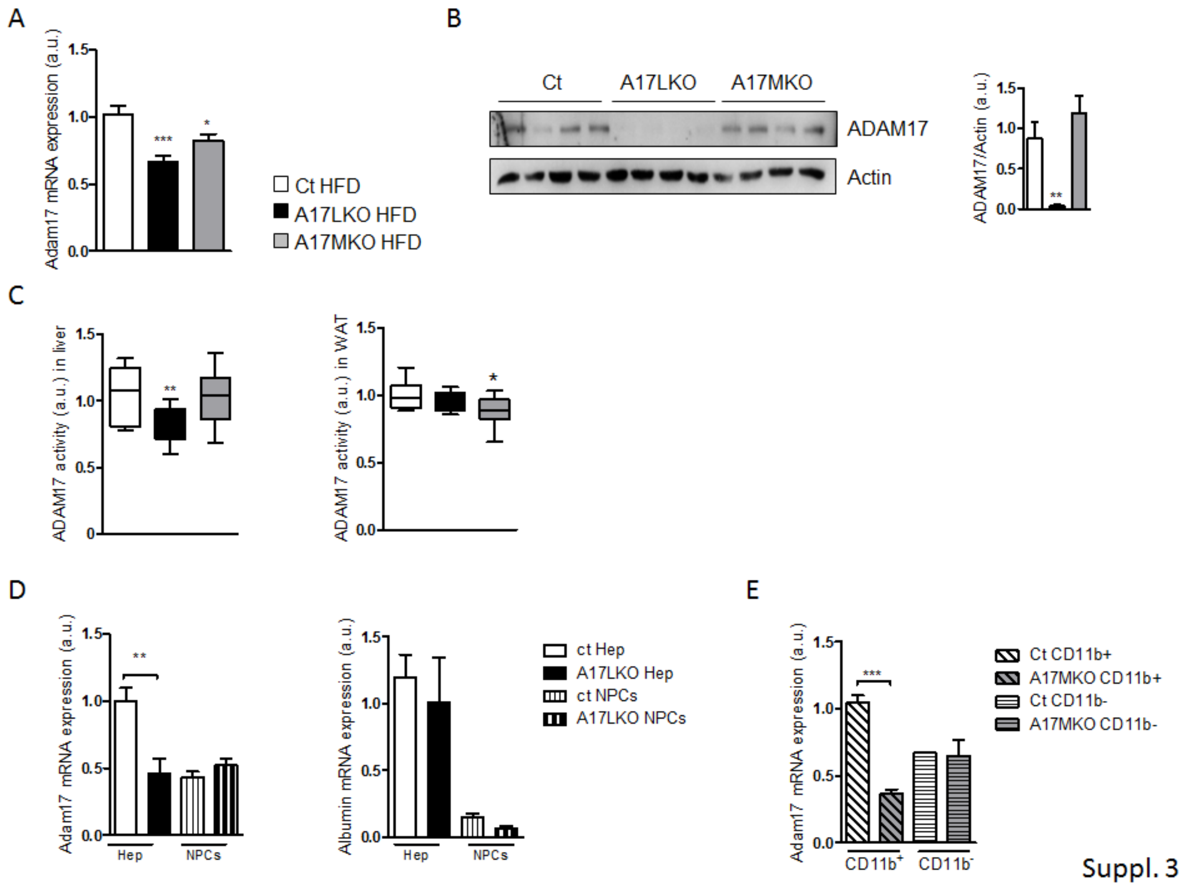
A
B
Ct
A17LKO
A17MKO
ADAM17
Actin
Adam17 mRNA expression (a.u.)
Ct HFD
A17LKO HFD
A17MKO HFD
ADAM17/Actin (a.u.)
C
ADAM17 activity (a.u.) in liver
ADAM17 activity (a.u.) in WAT
D
Adam17 mRNA expression (a.u.)
Albumin mRNA expression (a.u.)
Hep
NPCs
ct Hep
A17LKO Hep
ct NPCs
A17LKO NPCs
E
Adam17 mRNA expression (a.u.)
Ct CD11b+
A17MKO CD11b+
Ct CD11b-
A17MKO CD11b-
CD11b+
CD11b-

Suppl. 3

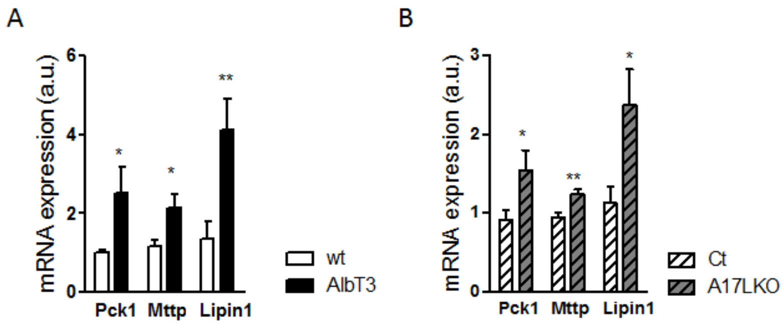
A
mRNA expression (a.u.)
0
2
4
6
*
*
**
Pck1
Mttp
Lipin1
wt
AlbT3
B
mRNA expression (a.u.)
0
1
2
3
*
**
*
Pck1
Mttp
Lipin1
Ct
A17LKO

Suppl. 4

4. Supplementary Full length Blots and Gels

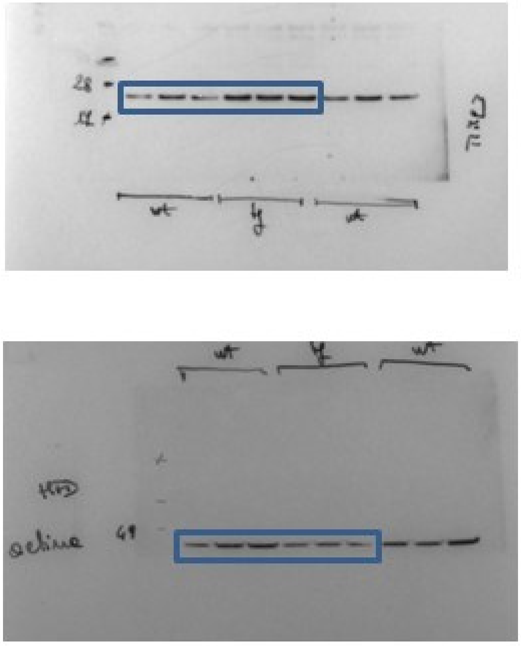

Fig2D

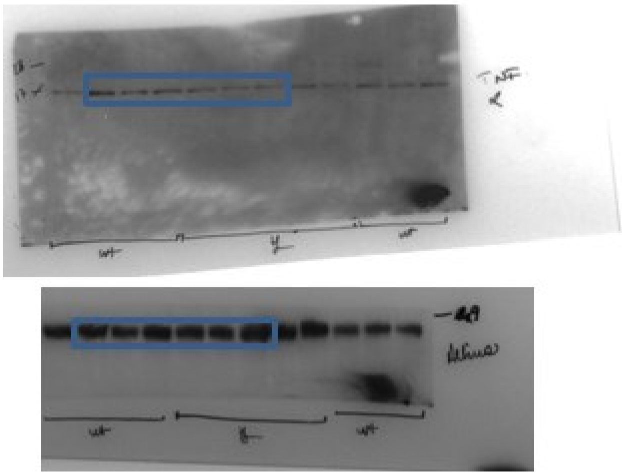

Fig2D

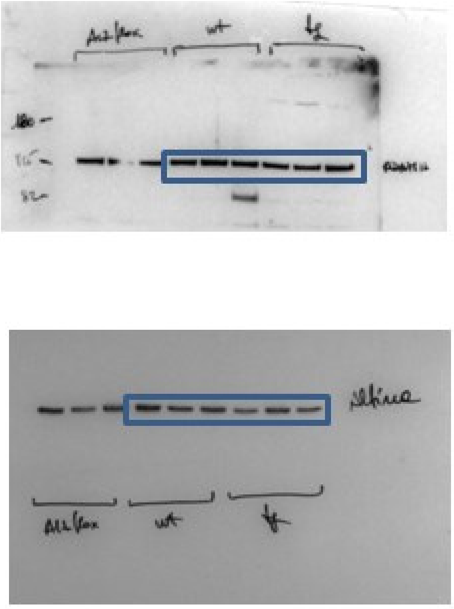

Fig2D

Liver Muscle WAT

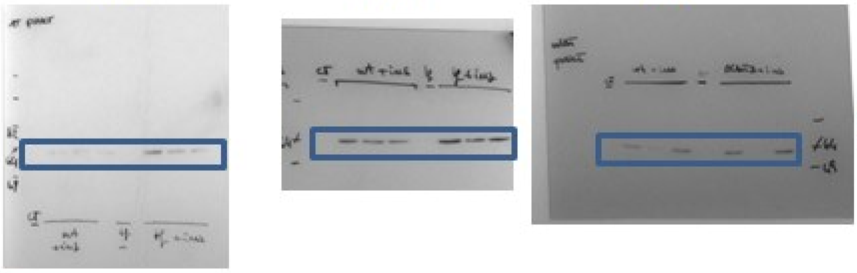

pSer473 Akt

Liver Muscle WAT

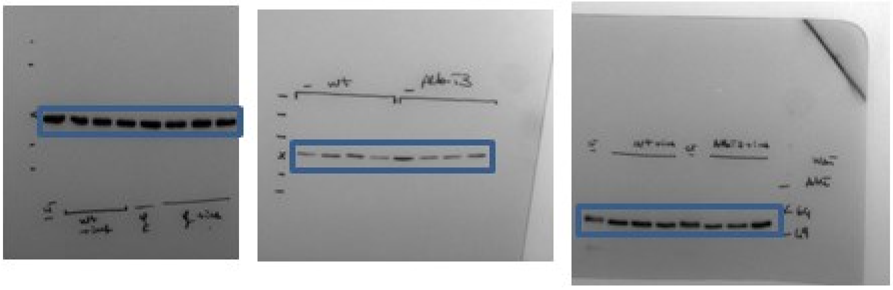

Akt

Fig2F

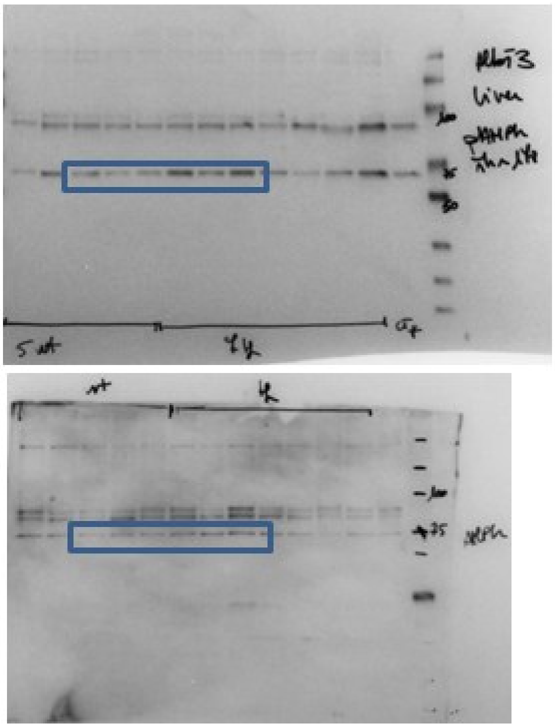

Fig3E

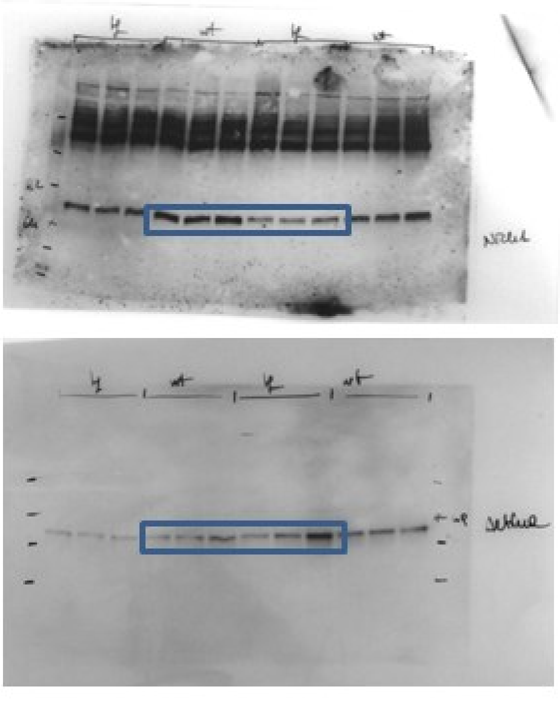

Fig4 F total extract

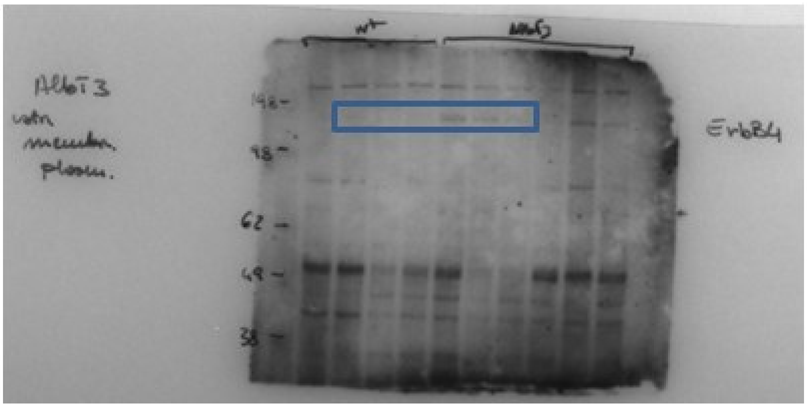

Ponceau S Staining

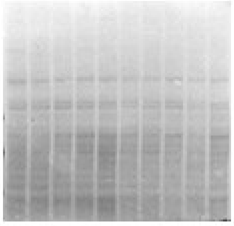

Fig4 F ErbB4
plasmatic
membrane extract

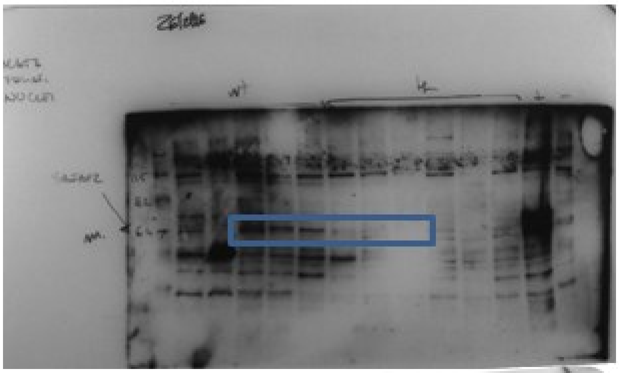

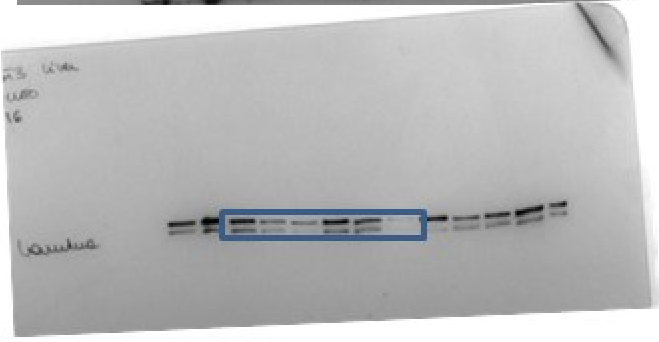

Fig4 F SREBP2
nuclear extract

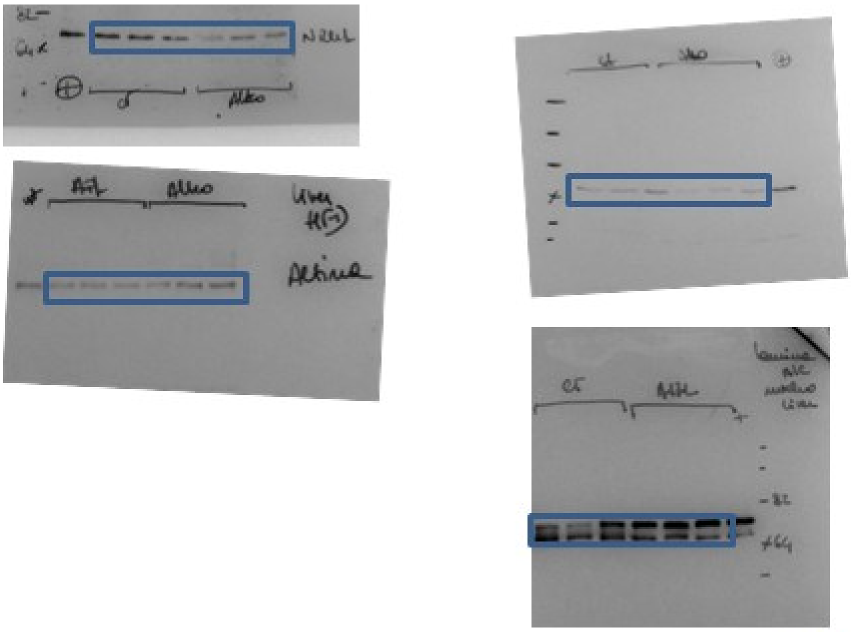

Fig6 C total and nuclear extract

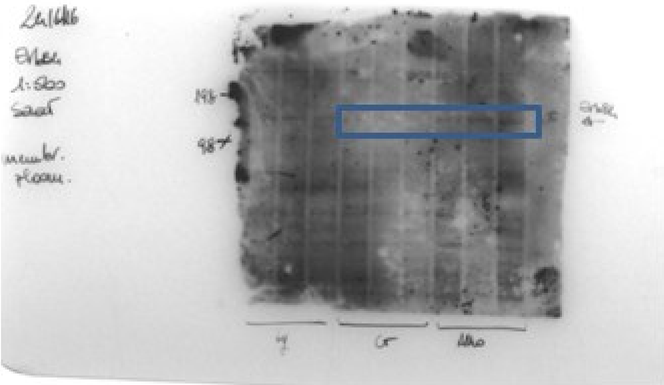

Ponceau S Staining

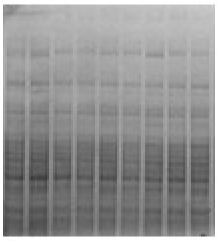

Fig6 C ErbB4 plasmatic membrane extract

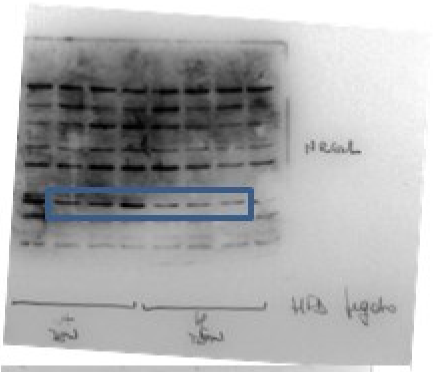
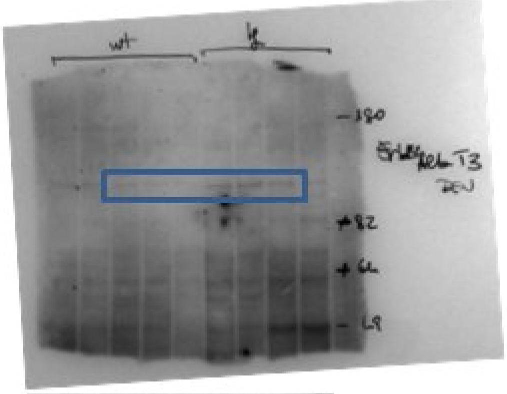
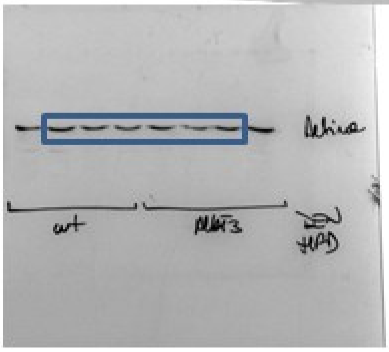
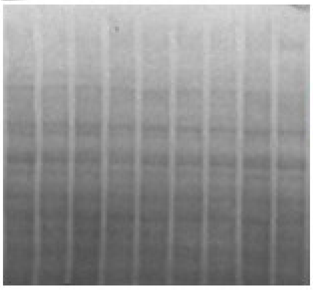

Ponceau S Staining

Fig 7 B, C

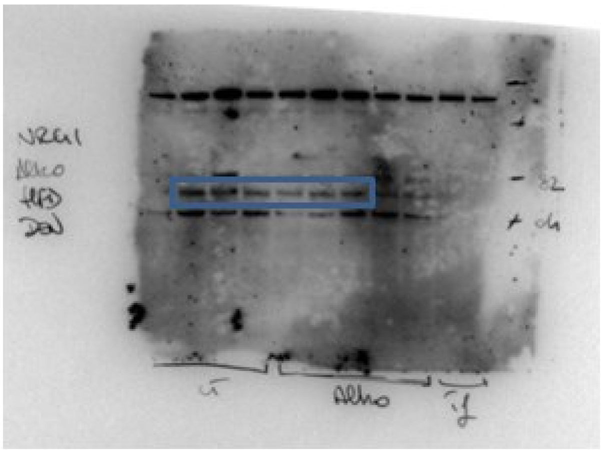

Fig 7 E

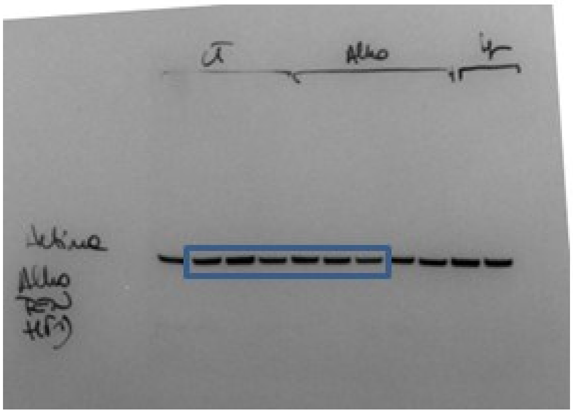

Fig 7 E

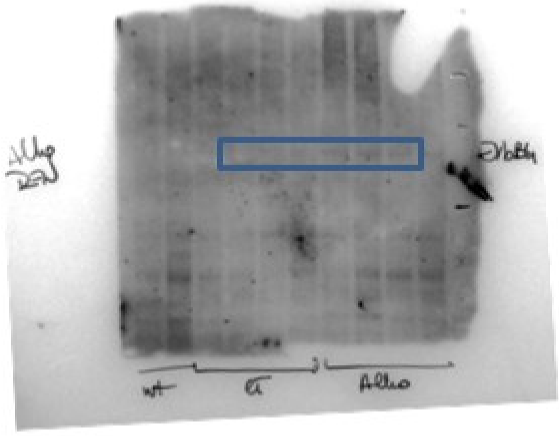

Ponceau S Staining

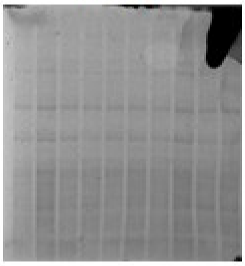

Fig 7 F

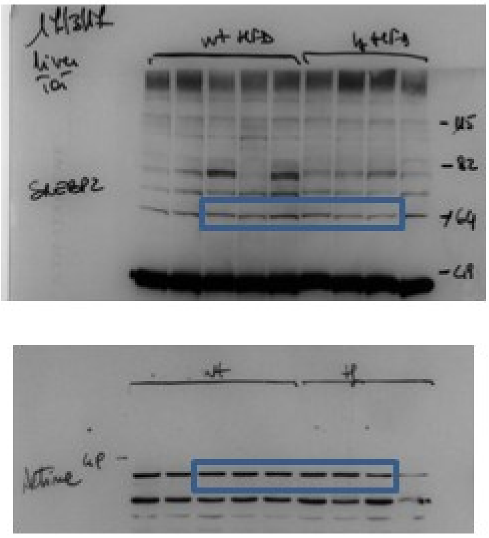

Suppl. Fig 2

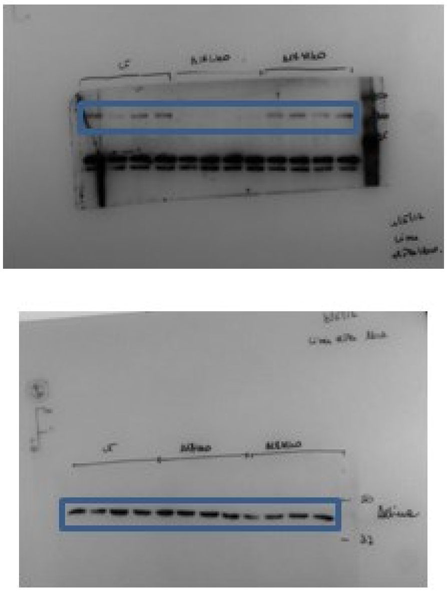

Suppl. Fig 3
